# Is depression a real risk factor for acute myocardial infarction mortality? A retrospective cohort study

**DOI:** 10.1186/s12888-019-2113-8

**Published:** 2019-04-24

**Authors:** Silvia Cocchio, Tatjana Baldovin, Patrizia Furlan, Alessandra Buja, Patrizia Casale, Marco Fonzo, Vincenzo Baldo, Chiara Bertoncello

**Affiliations:** 1 Department of Cardiac Thoracic Vascular Sciences and Public Health, Public Health Section, University of Padua, Via Loredan, 18, 35121 Padova, Italy; 2 Public Health Unit “Polesana”, Rovigo, Veneto Region Italy

**Keywords:** Epidemiology, Acute myocardial infarction, Depression, Survival

## Abstract

**Background:**

Depression has been associated with a higher risk of cardiovascular events and a higher mortality in patients with one or more comorbidities. This study investigated whether continuative use of antidepressants (ADs), considered as a proxy of a state of depression, prior to acute myocardial infarction (AMI) is associated with a higher mortality afterwards. The outcome to assess was mortality by AD use.

**Methods:**

A retrospective cohort study was conducted in the Veneto Region on hospital discharge records with a primary diagnosis of AMI in 2002–2015. Subsequent deaths were ascertained from mortality records. Drug purchases were used to identify AD users. A descriptive analysis was conducted on patients’ demographics and clinical data. Survival after discharge was assessed with a Kaplan-Meier survival analysis and Cox’s multiple regression model.

**Results:**

Among 3985 hospital discharge records considered, 349 (8.8%) patients were classified as ‘AD users’. The mean AMI-related hospitalization rate was 164.8/100,000 population/year, and declined significantly from 204.9 in 2002 to 130.0 in 2015, but only for AD users (− 40.4%). The mean overall follow-up was 4.6 ± 4.1 years. Overall, 523 patients (13.1%) died within 30 days of their AMI. The remainder survived a mean 5.3 ± 4.0 years. After adjusting for potential confounders, use of antidepressants was independently associated with mortality (adj OR = 1.75, 95% CI: 1.40–2.19).

**Conclusions:**

Our findings show that AD users hospitalized for AMI have a worse prognosis in terms of mortality. The use of routinely-available records can prove an efficient way to monitor trends in the state of health of specific subpopulations, enabling the early identification of AMI survivors with a history of antidepressant use.

## Background

Coronary heart disease (CHD) is the leading cause of mortality worldwide, and ranks among the top six causes of morbidity. Depression accounts for a relevant proportion of the global burden of disease, ranking among the top three causes, despite a low impact on mortality. In high-income countries, acute myocardial infarction (AMI) is the CHD carrying the highest mortality and morbidity rates [1–4].

In the USA, the age-adjusted hospitalization rates for CHDs decreased constantly between 2002 and 2013. Detailed data have shown a drop in the hospitalization rates for ST-segment elevation myocardial infarction (STEMI) and a rise in the proportion of hospital admissions for other forms (NSTEMI) in the past decade in both Europe and the United States [5–7].

A retrospective observational registry study conducted in Sweden found that the annual incidence rate and prevalence of depression rose steadily from 1991 to 2010, increasing more rapidly in women than in men [8]. For both genders, the incidence of clinically-relevant depressive symptoms increases with age, especially in the case of other ongoing comorbidities or institutionalization [9].

Following an episode of AMI, the incidence of depression ranges widely, from 15 to 30%, for major depressive disorder [10–12], and is around 20% for dysthymia (minor depression) or depressive symptoms [13]. Depression has been associated with a higher risk of cardiovascular events and a higher mortality in patients with one or more comorbidities [14]. Depression has been identified as a prognostic risk factor in CHD: the risk of all-cause mortality and the risk of cardiovascular events rise by 22 and 13%, respectively [3, 12]. Another study investigated the impact of depression on mortality after AMI, reporting a mortality risk at one year of 33% in patients previously diagnosed with depression, as opposed to 26% in the others; and at 19 years after the AMI, the mortality risk was 87 and 78%, respectively [15].

The occurrence of depression in patients with CHD substantially increases the likelihood of a worse cardiovascular prognosis [16]. Knowing the time frame of depression in relation to patient outcomes would have important mechanistic and screening implications [17]. Some authors reported that patients with and without a diagnosis of depression prior to their cardiac event had similar survival rates [18]. Others found mortality higher among patients with even mild, clinically insignificant depressive symptoms prior to their AMI than for those with no symptoms of depression [19]. A meta-analysis confirmed that both premorbid and postmorbid depression are of prognostic significance [17].

In the analysis of the fatality rate after hospitalization for AMI, another important factor to take into account is adherence to evidence-based treatment (EBT) for AMI patients after their discharge from hospital. The international guidelines recommend a combined and continuative use of beta-blockers, aspirin/clopidogrel, statins, and angiotensin-converting enzyme inhibitors or angiotensin receptor blockers (ACEIs/ARBs) after a myocardial infarction in order to reduce cardiac morbidity and mortality [20].

The objective of this study was to test the hypothesis of an association between a continuative use of antidepressants, as proxy of a state of depression, prior to the onset of AMI and a higher mortality afterwards, controlling for potential confounders.

## Methods

A retrospective cohort study was conducted using administrative data routinely collected by the Public Health District in the Province of Rovigo, in the Veneto Region of Italy. At the time of the study, the Public Health District served a population of about 173,000 with an average age of 45.3 years, an old-age dependency ratio of 193.7%, and a mortality rate of 11.2 per 1000 population.

### Sample

Patients were identified in the database on the basis of a linked anonymized personal code. Inclusion criteria were: hospitalization between December 1, 2002 and December 31, 2015, and discharge with a primary diagnosis of AMI, based on the International Classification of Diseases, Ninth Revision [ICD-9-CM] codes 410.xx. Patients were enrolled in the study only once, on their first hospitalization (i.e. they had not been admitted to hospital for AMI during the previous two years). The follow-up started 30 days after discharge and continued until time of death or 31/12/2015.

### Data sources and definition

Hospital discharge records, coded according to the ICD-9-CM, were extrapolated for all public and accredited private hospitals in the area. A specific ICD code (410.7x) was assigned to patients with NSTEMI, while other codes were used to define patients with STEMI. Deaths during the follow-up were ascertained from mortality records, coded according to the ICD- 9-CM, from the Veneto Cause of Death Register. For residents in the Public Health District of Rovigo the cause of death is coded, reported, and validated on death certificates by personnel at the local health agencies. We grouped the dead in five classes based on the the main cause of death: (i) malignancy (ICD: 140–239); (ii) endocrine diseases (ICD: 240–279); (iii) cardio-circulatory system illness (ICD: 390–459); (iv) respiratory illness (ICD: 460–519); and (v) others (all other ICD) [21].

Drug purchases, based on the Anatomical Therapeutic Chemical classification (ATC), were used to identify “users or non-users of antidepressants” and adherence to therapies.

To classify patients as users or non-users of antidepressants, the purchase of antidepressants was used as a proxy measure of a state of depression. Specifically, according to the ATC codes, the following classes of antidepressants were considered: non-selective monoamine reuptake inhibitors (NSMRIs) (ATC code: N06AA), selective serotonin reuptake inhibitors (SSRIs) (ATC code: N06AB), and other antidepressants (ATC code: N06AX). Patients were considered as users of antidepressants if they took this medication continuously for a period of at least 24 weeks within two years prior to admission for AMI.

As regards EBT for AMI, beta-blockers (ATC code: C07), aspirin /clopidogrel (ATC code: B01A), statins (ATC code: C10) and ACEIs/ARBs (ATC code: C09) were considered. After discharge, for each class of antidepressants and for each class of EBT for AMI, drug adherence was assessed by means of a medication possession ratio during the follow-up period, i.e. from the day after hospital discharge to the last day of involvement in the study (end of study or patient’s death). Specifically, for antidepressants and beta-blockers, statins and ACEIs/ARBs, we calculated the proportion of days on which a patient had the medication available (assuming that one pill a day was necessary), while aspirin/clopidogrel adherence was measured as the ratio of the number of dates on which drugs were prescribed to the number of months in the study. For each medication class, patients were classified as adherent (adherence measure > 80%) or nonadherent (otherwise). Patients were considered as adherent to the combined EBT for AMI if they had continuously taken medication from each of the four classes of drugs.

For all patients, comorbidities were identified from all secondary diagnoses in their hospital discharge records and graded according to the Charlson Comorbidity Index (CCI) [22], calculated as the sum of the scores for the severity of each comorbid condition, and then categorized as 0, 1, and 2 or more.

### Statistical analysis

A descriptive analysis was conducted on patients’ demographics and clinical data. Data were presented as percentages for categorical variables, and compared using the chi-square test or Fisher’s exact test, or as means ± standard deviations (SD), median with interquartile range (IQR) for continuous variables, which were compared using Student’s t-test for unpaired data, performing a priori tests for equality of variances. Baseline clinical characteristics were reported by age group as follows: up to 65 years old; from 66 to 80 years old; and 80 or older.

Patient survival after hospital discharge was assessed with a Kaplan-Meier survival analysis. Since it has been amply demonstrated that 30-day mortality is associated with AMI-related mortality, patients who died within 30 days of their AMI were excluded from the cumulative survival analysis. Survival curves were compared using the log-rank and Breslow tests. To examine the association between AD use and the outcomes of interest, we controlled for potential confounders (age, sex, CCI, type of AMI, and adherence to EBT for AMI and to antidepressant therapy after discharge) using a Cox’s multiple regression model. To assess the effect of the time variable on the risk of the event, and to overcome the hypothesis of intrinsic proportional risks in Cox’s model, the whole follow-up was divided into shorter time intervals, and a variable representing the period in which the event occurred was included in the regression model. A *p*-value of < 0.05 was accepted as statistically significant. The analyses were performed using the Statistical Package for the Social Sciences (SPSS 22.0; SPSS Inc., Chicago, USA). Significant trends in the study period of AMI-related hospitalization were assessed as average annual percent changes (AAPC) [23] using the Joinpoint Regression Program (JPR Version 4.7.0.0; Statistical Methodology and Applications Branch, Surveillance Research Program, National Cancer Institute, USA).

## Results

**Fig. 1:**
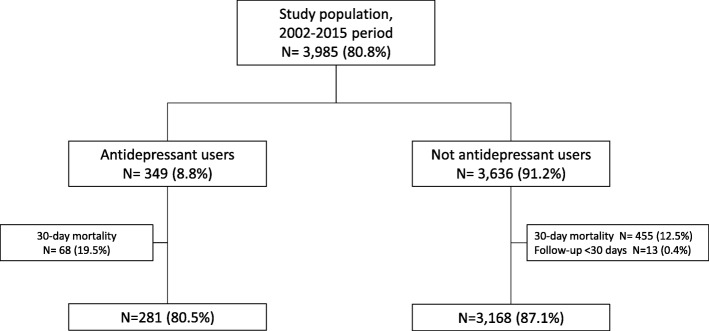
Flow-chart of selection process

During the study period, 3985 patients at their first hospitalization and with a primary diagnosis of AMI were considered. The flow-chart in Fig. 1 summarizes the patient and study group selection process.

Table 1 shows the study population’s demographic and baseline clinical characteristics. Overall, of the 3985 patients included in the analysis, 2428 (60.9%) were male. The men were significantly younger than the women (mean 68.2 ± 13.0 years versus 78.0 ± 12.0 years, *p* < 0.001). Among the 349 AD users, 48.7% used SSRIs, 20.9% used NSMRIs, and 30.4% used other ADs. The proportion of patients classified as “AD users” was significantly higher for women than for men (13.9% versus 5.4%, respectively, *p* < 0.001).

**Table 1:** Characteristics of 3985 patients hospitalized for AMI and stratified by AD use

Variables	Antidepressant use				*p* value
	Yes		No		
	(n. 349)		(n. 3636)		
Gender [*n*(%)]					
Males	132	(5.4)	2296	(94.6)	
Females	217	(13.9)	1340	(86.1)	*p* < 0.05
Age [mean ± (SD)]	76.1	(11.7)	71.6	(13.6)	*p* < 0.05
Follow-up in years [median ± (IQR)]	2.0	(0.2–5.2)	3.8	(0.8–7.7)	*p* < 0.05
Length of stay in days [median ± (IQR)]	10.0	(7.0–16.0)	10.0	(7.0–14.0)	*p* = 0.07
Charlson comorbidity index [n (%)]					
0	162	(8.0)	1868	(92.0)	
1	91	(8.5)	978	(91.5)	*p* = 0.60
≥ 2	96	(10.8)	790	(89.2)	*p* < 0.05
Acute myocardial infarction [*n*(%)]					
STEMI	199	(8.8)	2066	(91.2)	
NSTEMI	150	(8.7)	1570	(91.3)	*p* = 0.94

*SD* standard deviation, *IQR* interquartile range, *STEMI* ST-segment elevation myocardial infarction, *NSTEMI* non-ST-segment elevation myocardial infarction

Overall, considering a CCI of 1 ≥ 1, 49.1% of the hospital discharges (1955) involved patients with at least one comorbidity, with no significant difference between the groups of patients who were and were not AD users, but when a CCI ≥2 was considered, this was found significantly more often among the AD users (27.5% versus 21.7%, *p* < 0.05).

During the study period, the mean rate of AMI-related hospitalizations was 164.8 per 100,000 population a year (207.7 per 100,000 males, and 124.7 per 100,000 females). As concerns age, the peak was recorded among adults aged 80 years and over, with an AMI-related hospitalization rate of 8.4 per 1000 population a year. In the period considered, the temporal trend of AMI-related hospitalizations, and the AAPC in particular, showed a gradual and significant reduction from 204.9 in 2002 to 130.0 per 100,000 population in 2015, down 36.6% compared to 2002 [AAPC: − 2.7^(− 3.7; − 1.7)].

**Fig. 2:**
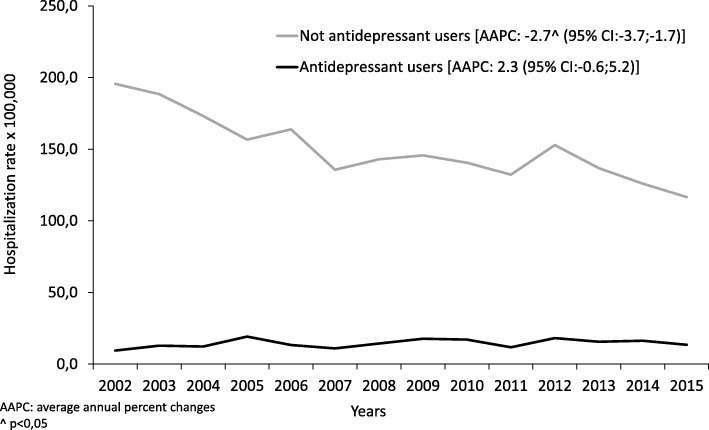
AMI-related hospitalization rate (per 100,000 population) by antidepressant use (2002–2015)

The temporal trends of the AMI-related hospitalization rates in patients stratified by AD users and non-users are shown in Fig. 2. The reduction in AMI-related hospitalizations was entirely attributable to the latter group, which saw a 40.4% reduction from 2002 to 2015 [AAPC: − 3.1 (95% CI:− 4.1; − 2.1)], whereas the trend among the AD users rose by 43.4% [AAPC: 2.3 (95% CI: 0.6; 5.2)].

The overall mean follow-up was 4.6 ± 4.1 years, while it was 3.2 ± 3.4 years for the AD users group and 4.7 ± 4.1 years for the other group. Overall, 523 subjects (13.1%) died within 30 days of their AMI, and for 89.3% of them the cause of death was cardiovascular disease.

**Fig. 3:**
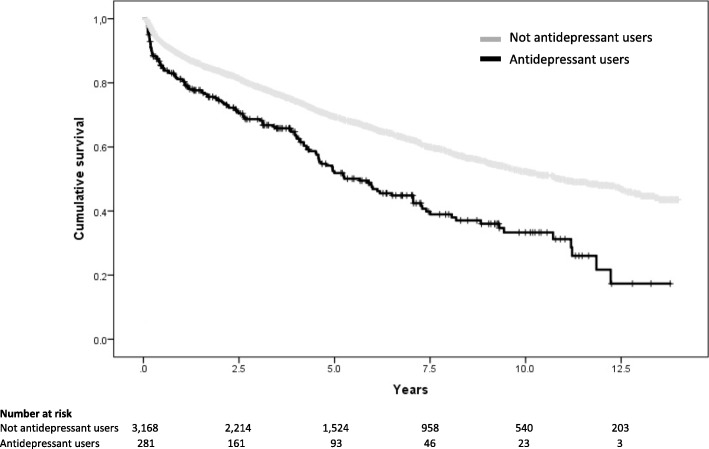
Survival analysis comparing AD users versus non-users

For the 3449 patients surviving beyond 30 days after their discharge from hospital, the mean survival was 5.3 ± 4.0 years. As shown in Fig. 3, the mean survival was shorter among AD users (4.0 ± 3.4 years) than among non-users (5.4 ± 4.0 years; *p* < 0.01).

During the follow-up, both all-cause mortality and cardiovascular-specific mortality were higher among AD users group than among non-users group (*p* < 0.01) (Table 2).

**Table 2:** Distribution of 1322 patients who died, by antidepressant use and cause of death

Cause of death	Antidepressant use				Total (n. 3449)	
	Yes (n. 281)		No (n. 3168)			
	*n*	(%)	*n*	(%)	*n*	(%)
Cardiocirculatory system illness	96	(34.2)	760	(24.0)	856	(24.8)
Malignancy	9	(3.2)	175	(5.5)	184	(5.3)
Endocrine diseases	3	(1.1)	63	(2.0)	66	(1.9)
Respiratory illness	4	(1.4)	49	(1.5)	53	(1.5)
Others	29	(10.3)	134	(4.2)	163	(4.7)
All deaths	141	(50.2)	1181	(37.3)	1322	(38.3)

Denominator of percentuage is the number of patients surviving beyond 30 days after their discharge from hospital (in the column)

Regarding their EBT for AMI, adherence was significantly higher - for each class of medication - among patients who were not AD users (*p* < 0.05). The aspirin/clopidogrel class of EBT for AMI was associated with the highest adherence in both groups, irrespective of any use of antidepressants. Considering the combined EBT for AMI, the overall adherence was 50.8%, and it was specifically 51.5% among not AD users, as opposed to 43.1% in the other group (*p* < 0.05) (Table 3).

**Table 3:** Adherence to AMI evidence-based treatment in the follow-up of 3449 patients

EBT class	Antidepressant use				Total (n. 3449)	
	Yes (n. 281)		No (n. 3168)			
	*n*	(%)	*n*	(%)	*n*	(%)
Aspirin/Clopidogrel	242	(86.1)	2879	(90.9)	3121	(90.5)
Beta-blockers	185	(65.8)	2308	(72.9)	2493	(72.3)
Statins	176	(62.6)	2293	(72.4)	2469	(71.6)
ACEIs/ARBs	184	(65.5)	2344	(74.0)	2528	(73.3)
Combination EBT	121	(43.1)	1630	(51.5)	1751	(50.8)

*EBT* evidence-based treatment, *ACEIs* angiotensin-converting enzyme inhibitors, *ARBs* angiotensin receptor blockers

After adjusting for potential confounders, the results confirmed that use of all three classes of antidepressants was independently associated with mortality: all ADs (adj OR = 1.75, 95% CI: 1.40–2.19); NMSRs (adj OR = 1.57, 95% CI: 1.10–2.29); SSRIs (adj OR = 1.71, 95% CI: 1.28–2.28): and other ADs (adj OR = 1.96, 95% CI: 1.42–2.72). Mortality was also significantly associated with sex, being higher among males (adj OR = 1.25, 95% CI: 1.12–1.41); age (adj OR = 1.06, 95% CI: 1.05–1.07); and CCI (adj OR = 1.19, 95% CI: 1.16–1.22). Adherence to combined EBT for AMI had a protective role (adj OR = 0.76, 95% CI: 0.73–0.80), while adherence to antidepressant treatment after discharge was irrelevant.

## Discussion

In our study, the AMI-related hospitalization rate decreased constantly during the period investigated, while among AD users the rate of hospitalization for AMI remained steady. A similar trend has been observed in other high-income countries, presumably due to a marked improvement in the treatment of CHDs in recent decades [24]. The steady rate of AMI-related hospitalization involving AD users could be explained by an improvement in the monitoring of antidepressant medication over the study period too, which may enable a better definition of depressive status.

The AMI-related hospitalization rate in our study was higher for men than for women; and males were younger than females on average. These results are consistent with the literature, which has drawn attention to gender-related differences in the occurrence of AMI [25–27].

About 9% of the patients in our sample had a history of antidepressant use, and they had a higher mortality after AMI than patients without this condition. Depressive disorders are common among people suffering from cardiovascular diseases and are associated with a broad range of adverse outcomes. In patients with AMI, depressive symptoms have emerged as a prognostic risk factor for a higher cardiovascular-specific mortality, recurrent hospital admissions, worse general health and well-being, and higher costs of care, even in the absence of a clinical diagnosis of depression [28, 29]. The women in our sample were more likely to use antidepressants before being hospitalized for AMI. This finding is consistent with a multicenter study conducted in Canada, the USA and Switzerland, in which data were collected on patients aged 18–55 years using a self-report questionnaire that included a validated standard tool for assessing depression [30–33].

Regarding the association between antidepressant use and the risk of CHD among subjects with no history of CHD, the results of a meta-analysis provide no evidence of any association between SSRI or NSMRI use and CHD risk [34]. Although the use of antidepressants in patients with CHD helps to control their depression, their use in this group is controversial. The results of a meta-analysis nonetheless found that SSRI use in these patients reduces their depression symptoms and may improve their CHD prognosis [16].

Patients hospitalized with AMI are at particularly high risk of mortality, but the mortality rates for patients hospitalized with heart failure can vary significantly, ranging from 5 to 9% [35]. The prognosis after hospitalization for AMI is also reportedly very poor, with a 10-year mortality risk of 48.6% [36]. The 30-day mortality rate of 13.1% seen in our sample could be due to the patients’ advanced age, and associated chronic conditions. In fact, the sample’s long-term survival was consistent with other published reports.

The association seen between chronic physical conditions and depression is common. Depression has been associated with a higher incidence of at least one chronic comorbidity, while people with chronic diseases have been found more prone to suffer from depression than people in good health [37, 38]. Our study identified a better survival rate for patients hospitalized for AMI with no history of antidepressant treatment. This difference, was confirmed even after adjusting for age, gender, CCI, adherence to EBT for AMI, and type of AMI. Depression in patients who experience AMI remains a severe condition and warrants treatment and care. Patients with a history of moderate-severe depressive symptoms may benefit from adequate treatment, or at least careful clinical follow-up, after they have been hospitalized for AMI, in order to monitor any rapid mood swings and start treatment should symptoms persist.

Patients with depression are less likely to adopt an adequately healthy lifestyle in order to reduce their cardiac risk after an AMI [39], and nonadherence to medication is significantly higher among the depressed [40]. Focusing on the evidence-based treatment as a whole, the proportions of patients adhering to the combined EBT for AMI were much the same as in other studies, and our findings confirm the positive effects of EBT for AMI on survival after AMI [20].

Our results show that it is important to identify AMI patients at high risk of depression, so that they can be targeted for depression screening, and benefit from appropriate treatment in relation to their baseline conditions [18]. In our study, there was no difference in mortality between patients who were or were not given continuative antidepressants after discharge. This finding is consistent with another study that examined the timing of antidepressant prescription vis-à-vis the time of hospital discharge after AMI [19]. It is still by no means clear whether managing any depression can improve survival after AMI, and clinical trials on this issue are obviously needed, also to ascertain the most appropriate treatment for post-AMI depression. Currently-available evidence nonetheless indicates that psychotherapy and psychoactive pharmacological treatments are both safe and effective in reducing depression in patients with cardiovascular diseases [41].

A limitation of our study lies in that patients were identified as AD users (considered as a proxy for a state of depression) on the basis of their having purchased antidepressants for more than 24 consecutive weeks prior to their hospitalization for AMI, not on the grounds of a clinical diagnosis. The threshold of 24 weeks has been adopted by several clinical practice guidelines as an indication of treatment for chronically depressed patients, however [42]. A clinical assessment of the severity of AMI was also lacking in our study. The main strength of our study, on the other hand, concerns the AMI population analyzed, which was numerically large, well defined, and followed up for several years after discharge from hospital.

## Conclusions

Our findings show that AD users hospitalized for AMI have a worse prognosis in terms of mortality. The use of routinely-available records can prove an efficient way to monitor trends in the state of health of specific subpopulations, enabling the early identification of AMI survivors with a history of depression.

## Abbreviations

AAPC: Average annual percent changes
ACEIs: Angiotensin-converting enzyme inhibitors
ADs: Antidepressants
AMI: Acute myocardial infarction
APC: Annual percent change
ARBs: Angiotensin receptor blockers
ATC: Anatomical therapeutic chemical classification system
CCI: Charlson Comorbidity Index
CHD: Coronary heart disease
EBT: Evidence-based treatment
ICD-9-CM: International Classification of Diseases, Ninth Revision
IQR: Interquartile range
NSMRIs: Non-selective monoamine reuptake inhibitors
NSTEMI: Non-ST-segment elevation myocardial infarction
SSRIs: Selective serotonin reuptake inhibitors
STEMI: ST-segment elevation myocardial infarction
